# Drug repositioning in thyroid cancer: from point mutations to gene fusions

**DOI:** 10.3389/fonc.2024.1407511

**Published:** 2024-05-08

**Authors:** David Sánchez-Marín, Macrina Beatriz Silva-Cázares, Manuel González-Del Carmen, Alma D. Campos-Parra

**Affiliations:** ^1^ Posgrado en Ciencias Biológicas, Facultad de Medicina, Universidad Nacional Autónoma de Mexico (UNAM), Ciudad de Mexico, Mexico; ^2^ Unidad Académica Multidisciplinaria Región Altiplano, Universidad Autónoma de San Luis Potosí, (UASL), Matehuala, San Luis Potosí, Mexico; ^3^ Facultad de Medicina, Universidad Veracruzana (UV), Ciudad Mendoza, Veracruz, Mexico; ^4^ Instituto de Salud Pública, Universidad Veracruzana (UV), Xalapa, Veracruz, Mexico

**Keywords:** thyroid cancer, variants, repurposed drugs, gene fusions, mutations

## Abstract

The diagnosis of thyroid cancer (TC) has increased dramatically in recent years. Papillary TC is the most frequent type and has shown a good prognosis. Conventional treatments for TC are surgery, hormonal therapy, radioactive iodine, chemotherapy, and targeted therapy. However, resistance to treatments is well documented in almost 20% of all cases. Genomic sequencing has provided valuable information to help identify variants that hinder the success of chemotherapy as well as to determine which of those represent potentially druggable targets. There is a plethora of targeted therapies for cancer, most of them directed toward point mutations; however, chromosomal rearrangements that generate fusion genes are becoming relevant in cancer but have been less explored in TC. Therefore, it is relevant to identify new potential inhibitors for genes that are recurrent in the formation of gene fusions. In this review, we focus on describing potentially druggable variants and propose both point variants and fusion genes as targets for drug repositioning in TC.

## Introduction

Thyroid cancer (TC) is the most common malignant tumor of the endocrine system, with 586,202 new cases worldwide in 2020 (1). The overall incidence of TC has increased dramatically in the last 30 years. This increase may be due to overdiagnosis, thanks to improvements made in diagnostic procedures (2). Morphologically and clinically, TC is classified into two main groups: differentiated cancer—comprising papillary and follicular thyroid cancer—and undifferentiated TC, designated anaplastic carcinoma of the thyroid (3). The most prevalent is papillary thyroid cancer (PTC), which accounts for up to 85% and has a good prognosis (5-year survival rate of more than 95%, mainly in patients with stage I or II disease), as does follicular thyroid cancer (FTC), which is less prevalent, accounting for 15% of all cases (4). Patients with poorly differentiated or anaplastic TC, advanced-stage disease, or distant metastases have higher mortality rates (5). Moreover, about 20% of PTC patients manifest disease recurrence because of drug resistance, suggesting a change in treatment approaches. This points out the need to personalize treatments, including drug repositioning (6). Target therapy can be repositioned and offers greater success since it can be customized according to the patient’s genomic alterations. In this review, we highlight therapeutic opportunities for TC, focusing on druggable genes with potential repositioning for personalized therapy.

## Classical point mutations in thyroid cancer: windows of opportunity for the use of drug repositioning

**Figure 1:**
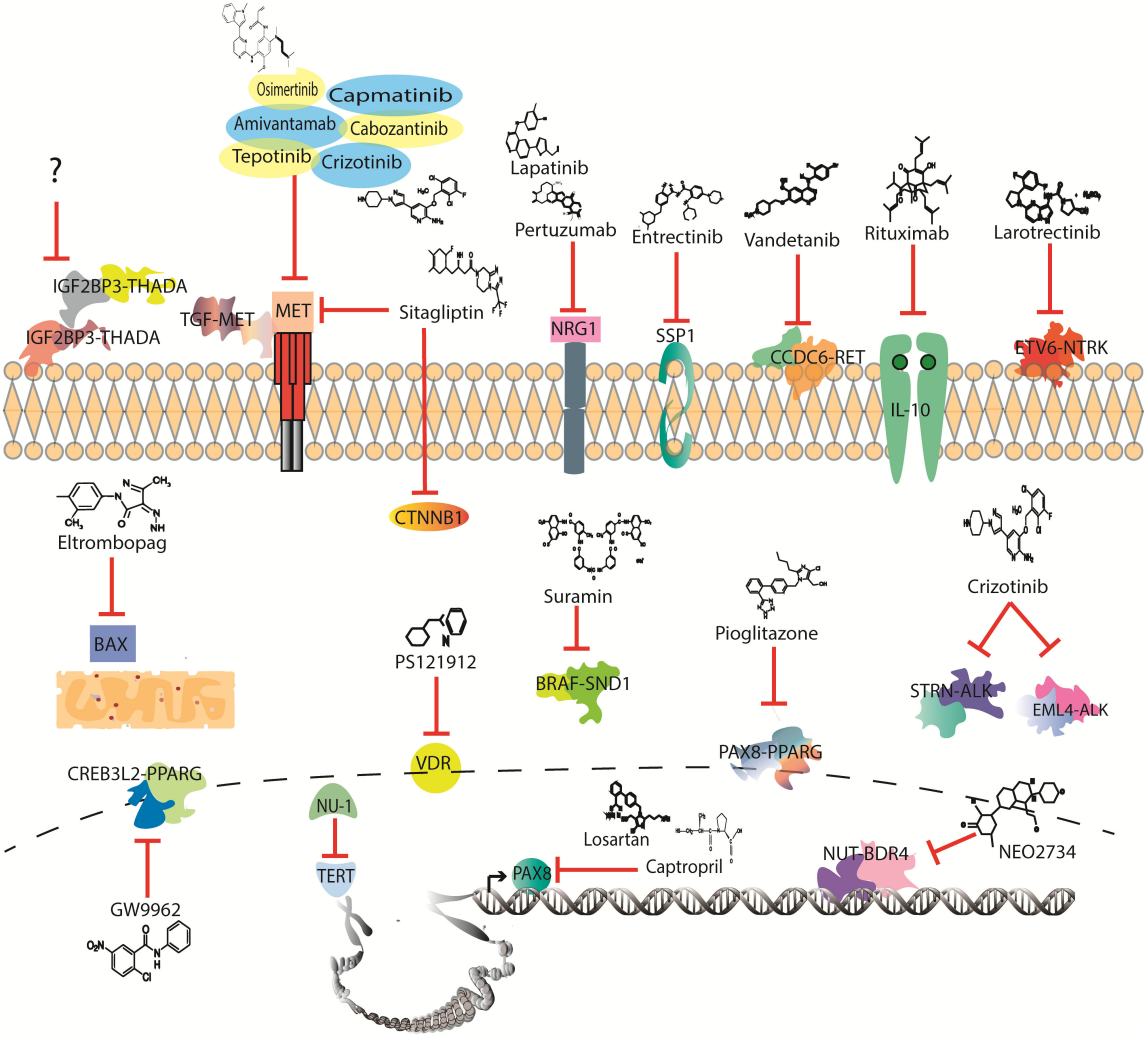
Drugs with potential to be investigated in thyroid cancer clinical trials according to mutational profile.

Radioactive iodine administration and/or surgery remain the first line of treatment for TC; however, for advanced disease, chemotherapy (CT) becomes the systemic option of treatment available (7). Nevertheless, CT constantly faces resistance and severe secondary effects (8). Therefore, it is necessary to overcome resistance by recognizing drug-susceptible mutations, which may lead to the identification of a broad spectrum of target therapies that could be repositioned in TC (**Figure 1**).

Next-Generation Sequencing (NGS) has made it possible to sequence the genomes of different types of cancer, which has revealed that around 90% of patients with TC have one or more genetic abnormalities (9). Dysregulation of phosphatidylinositol 3-kinase (*PI3KCA*) and mitogen-activated protein kinase (*MAPK*) signaling pathways is mainly affected by point mutations in target genes such as B-Raf proto-oncogene, serine/threonine kinase (*BRAF*), A-Raf proto-oncogene, serine/threonine kinase (*RAS*), and ret proto-oncogene (*RET*) (10). One of the best-documented and highest prevalence point mutations in PTC is *BRAF* exon 15 p. V600E (45% of all cases), which is associated with poor prognosis and high recurrence (11). The *BRAF* exon 15 p. V600E variant has constitutively active *BRAF* serine–threonine leading to the activation of effectors of the *MAPK* pathway and, consequently, surveillance and proliferation (11). Vemurafenib has shown antitumor activity in patients with *BRAF* exon 15 p. V600E-positive progressive PTC, representing a potential new therapeutic option (12, 13). Ipilimumab, nivolumab, dabrafenib, and trametinib are also approved target therapy options for *BRAF* mutations in melanoma (14) that could be repositioned to TC. In addition, drugs blocking phosphatase and tensin homolog (*PTEN*) and *PI3KCA* homogenize the font of the letter with that of the rest of the text effects (**Table 1**).

**Table 1:** Variants in potentially druggable genes in thyroid cancer.

Drug	Gene	Variant in thyroid cancer	Cancer	Reference
Ipilimumab, nivolumab, dabrafenib, and trametinib	BRAF	V600E	Melanoma	(14)
Alpelisib	PI3KCA	GAA>AAA, G1564A/CCA>TCA, C3031T	Breast	(15)
Sotorasib and adagrasib	KRAS	G12C	NSCL	(16)
Vandenitib and cabozantinib	RET	Codons: 609, 611, 618, and 620 Val804Met. S836S	NSCL	(17)
Amivantamab, cabozantinib, capmatinib, crizotinib, osimertinib, tepotinib, and sitagliptin	MET	rs1621 AG	NSCL, thyroid	(18) (19–23), (18)
Sitagliptin	CTNNB1	c.133T>C	Thyroid	(24)
Afatinib, lapatinib, and pertuzumab	NRG1	rs2439302	Colorectal, breast	(25)
Entrectinib	SPP1	rs4754	Cervical	(26)
Eltrombopag	BAX	−248 G>A	–––	(27)
PS121912	VDR	(rs2228570) CT/TT	Leukemia	(28)
Rituximab	IL-10	G-1082A	B-cell non-Hodgkin’s lymphoma	(29)
Nu-1	TERT	C>T (C228T) and 1,295,250 C>T (C250T)	Lung and colorectal	(30)
Vandetanib	CCDC6-RET	Gene fusion	NSCL	(17)
Larotrectinib	ETV6-NTRK3	Gene fusion	Secretory breast cancer	(31)
Crizotinib, ceritinib, alectinib, brigatinib, and lorlatinib	STRN-ALK	Gene fusion	Lung adenocarcinoma	(32)
Crizotinib, ceritinib, alectinib, brigatinib, and lorlatinib	EML4-ALK	Gene fusion	Lung	(33)
Suramin	BRAF-SND1	Gene fusion	–––	(33)
–––	IGF2BP3-THADA	Gene fusion	–––	(34)
Pioglitazone, GW9662	PAX8-PPARG	Gene fusion	Thyroid	(35)
NEO2734	NUT-BRD4	Gene fusion	NUT midline carcinoma	(36)
Amivantamab, cabozantinib, capmatinib, crizotinib, osimertinib, tepotinib, and sitagliptin	TGF-MET	Gene fusion	Sarcoma, glioma	(37)

*PI3KCA* is another gene with several missense mutations in three subtypes of TC: follicular, papillary, and anaplastic. Interestingly, *PI3KC*A mutations are associated with drug resistance in *BRA*F exon 15 p. V600E-positive cases. In this scenario, it is worth looking at how alpelisib can counteract the resistance mechanism by diminishing the EPH receptor B2 (*EPHB2*)-induced signaling (38). Consistent with the latest, *PTEN*, which has a negative regulatory role in the same pathway, has reported variants in TC (39).

KRAS proto-oncogene, GTPase (*KRAS*), is a G protein that plays an important role in the *PI3KCA*/*MAPK* signaling pathway. Point mutations in *KRAS* usually occur at codons 12, 13, and 61 and have been found in FTC and PTC at a frequency of 50% and 20%, respectively. These mutations confer a more aggressive phenotype and increase the risk of mortality (40). Sotorasib and adagrasib are *KRAS* exon 2 p. G12C mutation drugs approved for non-small cell lung cancer (NSCLC) (16, 41). It remains of interest to analyze the effect of these drugs on TC harboring the *KRAS* exon 2 p. G12C mutation. *RET* is another gene commonly mutated in PTC and medullary thyroid cancer (MTC), with both large rearrangements and point mutations reported. *RET* is a receptor tyrosine kinase that regulates growth, survival, migration, and survival, activating multiple intracellular signaling pathways, including *PI3KCA/AKT* serine/threonine kinase 1 (*AKT*), *MAPK*, mitogen-activated protein kinase 8 (*JNK*), and others. Oncogenic activating point mutations can occur mainly in the extracellular domain, particularly in codon C634 of exon 11, in 609, 611, 618, or 620 of exon 10, and in M918 of exon 16, being *RET* exon 16 p. M918T mutation the most common and represents more than 75% of all *RET* somatic mutations found in MTC (42). Selpercatinib and pralsetinib are *RET*-specific inhibitors approved for the MTC variant and have been well tolerated (43–45). Other multitargeted kinase inhibitors used to inhibit the *PI3KCA/AKT*/mechanistic target of the rapamycin kinase (*MTOR*) pathway in MTC are vandenitib and cabozantinib. The first one inhibits *RET* but also inhibits other kinases such as vascular endothelial growth factor receptor 2 (*VEGFR2*), vascular endothelial growth factor receptor 3 (*VEGFR3*), and epidermal growth factor receptor (*EGFR*), while cabozantinib inhibits *RET*, vascular endothelial growth factor (*VEGF*), *MET* proto-oncogene, receptor tyrosine kinase (*MET*), and *ROS* proto-oncogene 1, receptor tyrosine kinase (*ROS1*) (46). Both inhibitors have shown efficacy and improved overall survival in patients harboring *RET* exon 16 p. M918T mutation (47, 48).

*MET* is a receptor tyrosine kinase that has an oncogene role in promoting angiogenesis due to downstream activation of *RAS*, *PI3KCA*, and signal transducer and activator of transcription 3 (*STAT3*) signaling pathways (49). Drugs that inhibit *MET* are amivantamab, cabozantinib, capmatinib, crizotinib, osimertinib, and tepotinib (50). Particularly, *MET* has a reported variant in NSCLC that skips the exon 14 and makes the protein constitutively active (51). In TC, it constitutes an inclusion criterion for thyroid gland medullary carcinoma (52, 53). Currently, therapy targeting *MET*, although only indicated to treat NSCL, represents a potential target in TC. Furthermore, a PTC expression signature has been identified in which three genes are overexpressed, promoting metastasis and being associated with poor prognosis: dipeptidyl peptidase 4 (*DPP4*), *MET*, and catenin beta 1 (*CTNNB1*). The signature is associated with immunosuppression and correlates with tumor infiltration of tumor-associated macrophages, which leads to T-cell exclusion. Interestingly, sitagliptin, an FDA-approved drug to treat diabetes type II, has affinity not only to *DPP4* (diabetes target) but also to *MET* and *CTNNB1* (54–56). Moreover, the affinity for *MET* and *CTNNB1* is even higher than FDA-approved inhibitors specific for each of them, like crizotinib and PNU-74654, respectively. Therefore, sitagliptin represents a multidrug therapy window for TC (18).

Paired box 8 (*PAX8*), a gene implicated in proliferation and migration, is usually overexpressed in TC. Likewise, in high-grade serous ovarian cancer, *PAX8* is upregulated (57). Remarkably, losartan and captopril, which are FDA-approved drugs, have been found effective at inhibiting *PAX8* expression and function. This evidence suggests potential therapeutic opportunities using losartan and captopril, not only for ovarian cancer but also for TC (57).

Besides the variants reported in the above-mentioned genes, there are also polymorphisms associated with TC (58). For instance, neuregulin 1 (*NRG1*) acts as an oncogene through its role as a glycoprotein that mediates cell-to-cell signaling (59). In breast cancer, lapatinib may be used to inhibit *EGFR* and erb-b2 receptor tyrosine kinase 2 (*HER2*) kinases, two receptors of also relevant function in TC. Nonetheless, resistance is acquired and correlates with an increased expression of *NRG1*. By trying to overcome it, adding pertuzumab has shown promising results in decreasing *NRG1*-acquired resistance and tumor progression (25).

Similarly, secreted phosphoprotein 1 (*SPP1*), an integrin-binding glycophosphoprotein overexpressed in TC that promotes tumorigenesis through the inhibition of differentiation factors of thyroid cells, represents an opportunity for drug repositioning (60, 61). Although there are no current FDA drugs approved for inhibiting *SPP1*, a recent publication showed a promising inhibitory drug for cervical cancer: entrectinib (26). This represents a highlight, as entrectinib is an FDA-approved drug for NTRK fusions in solid tumors, including TC (62).

As with *SPP1*, another window of opportunity for targeted treatment is *BCL2*-associated X, apoptosis regulator (*BAX*). This gene participates in mitochondrial regulation of cell death; however, in cancer, it contributes to cell death dysregulation (63). Importantly, in TC, *BAX* has a reported polymorphism positively correlated with PTC, and more importantly, the FDA-approved drug eltrombopag acts as a *BAX* inhibitor, which drives apoptosis induction (64, 65). *SPP1* and *BAX* are not the only genes in which polymorphism is related to TC. *VDR* stands for vitamin D receptor and has been associated with cancer development (66). It is not well established if TC contributes to or attenuates tumor growth; however, two polymorphisms, FokI and TaqI, are associated with a more aggressive type, and the heterozygous FokI to metastasis (67). Remarkably, it has been shown that antagonists of vitamin D have therapeutic effects as they inhibit downstream cell cycle proliferation. There is already an insight into potential therapies using *VDR* as a druggable target. For instance, PS121912 has shown promising therapeutic effects by acting as a selective *VDR* inhibitor (28).

In the immunology context, several profiles have been described causing differential expression and immune cell proliferation among TC subtypes (68). Interleukin-10 (*IL-10*) is one of several dysregulated cytokines in TC associated with immunological and apoptosis evasion and aggressiveness (68). This effect is caused by expression induction of *BCL2* like 1 (*bcl-xL*) and *BCL2* apoptosis regulator (*BcCL2*) and resistance to *CD95*-mediated apoptosis (69, 70). Due to its oncological role, *IL-10* figures as a potential therapeutic target. Rituximab has promising inhibitory effects against *IL-10* through downregulation of *BCL2* and sensitization of B-cell non-Hodgkin’s lymphoma to apoptosis (29). However, resistance constitutes a problem due to broad kinase inhibitor activity and toxicity, which may limit their use and encourage the use of more specific inhibitors (71).

Lastly, telomerase reverse transcriptase (*TERT*), an enzyme known to be implicated in cancer, has been described as one of the most frequently mutated genes in TC, particularly in its promoter, which causes its overactivation. *TERT* inhibitors are currently under study, and NU-1 not only sensitizes the cell to chemo- and radiotherapy but also can inhibit proliferation and increase immune activity (30).

From NGS of long DNA fragments, gene fusions have been identified. When two genes conform to a fusion, they either lose or gain function. In cancer, they can contribute to tumor growth due to constitutive activation of an oncogene, such as *BCR-ABL* (72). Remarkably, some gene fusions are considered drivers, while others contribute to generating more genomic instability and disease development. There are gene fusions that are found across various cancers (73). These features of gene fusions represent an unprecedented opportunity to develop target therapies aimed at providing personalized medicine to patients.

## Spotlight of novel therapies: gene fusions

Over 50 gene fusions have been identified in TC, which are mainly conformed by the *RET*, neurotrophic receptor tyrosine kinase (*NTRK*), *ALK*, and *BRAF* genes (74). These genes are tyrosine kinase overactivated mainly due to kinase retention and overexpression by transcription factors of the parental genes, making them druggable targets (75). Currently, three drugs are being used in clinics to treat TC-targeting gene fusions: pralsetinib, selpercatinib, and larotrectinib (76). The first two are *RET* inhibitors and were first set as a treatment for both point mutations and gene fusions; however, selpercatinib shows efficacy in specific *RET* variant genotypes that present pralsetinib resistance. For instance, *BaF4/KIF5B-RET* shows tumor growth despite treatment with pralsetinib, while selpercatinib can effectively inhibit growth (44, 77). However, as with other variants, these gene fusions are not expressed across all subtypes of cancer, while some therapies face drug resistance and lack of treatment for greater, yet untargeted variants (**Figure 1**).

For *RET*, 19 fusions have been described; however, only therapies consisting of *RET* inhibitors are currently available, leaving the partner genes pharmacologically unexplored (78). This is of great importance as it has been described that the inhibitory sensitivity of several gene fusions varies depending on the partner gene; hence, drug screening should be performed testing not only the most common gene. For instance, the coiled-coil domain containing 6 (*CCDC6*) is a recurrent gene-forming fusion with *RET* in lung cancer, where it has shown potential druggability of *EGFR* inhibitors in combination with *RET* inhibitors, decreasing resistance to *RET* inhibitors while also enhancing sensitivity to *PARP* inhibitors (79). Particularly, the fusion *CCDC6-RET* is more sensitive to vandetanib due to the off-target inhibitory effect and crosstalk with *EGFR* pathway activation (80). Furthermore, this fusion and *ERC1-RET* have not had a response to the *RET* drug, cabozantinib, supporting the idea of focusing on the second gene as well (81).

Larotrectinib targets the *NTRK* genes, which are neurotrophic tyrosine kinase receptors. If binding occurs, the protein phosphorylates itself and activates the *MAPK* pathway. Therefore, as part of a gene fusion, it causes its constitutive activation (82). Several fusions involving *NTRK1*, *NTRK2*, and *NTRK3* have been described in the lung, colon, brain, head and neck, and TC (83). For this reason, it has been a promising targeted therapy, as the same fusions can occur in several tissues. In TC, larotrectinib is administered to patients diagnosed with the anaplastic subtype, and tumor growth continues despite other treatments (82). An example of this is the *ETV6-NTRK3* fusion, which has been described as a driver variant in secretory breast cancer with high efficacy upon larotrectinib treatment (84). However, larotrectinib therapy targets only the *NTRK* gene, while their partner genes remain untargeted. For instance, sequestosome 1 (*SQSTM1)* is a gene that conforms to fusions with both *NTRKs* and plays a role in autophagy, specifically through the *AKT*/protein kinase AMP-activated catalytic subunit alpha 2 (*AMPK*)/*MTOR* signaling reported in PTC (84).

Although only three drugs are being used in TC to target gene fusions, there are several other recurrent genes forming gene fusions that are already targeted in other cancers. On one hand, there is *ALK*, which is widely known for its oncogenic role, especially as part of gene fusions (85). Currently, *ALK* fusions do not have a regimen of treatment for TC, but its potential has already been evaluated. For example, *STRN-ALK* and *EML4-ALK* are promising targets in TC using the FDA-approved drug crizotinib, among other drugs such as ceritinib, alectinib, brigatinib, and lorlatinib (32, 33).

On the other hand, there are *BRAF* fusions, and remarkably, despite *BRAF* having several target drugs, none of them are used to treat TC. Furthermore, among all the gene partners of *BRAF*, staphylococcal nuclease and Tudor domain containing 1 (*SND1*), an oncogene in several types of cancer acts in addition to posttranscriptional modifications (86). This is a highlight for novel therapy, as a small molecule called suramin has been identified to inhibit their protein by impairing its interaction with several microRNAs and sensitizing the response to standard chemotherapy (87).

Interestingly, up to five gene fusions are involved in *THADA* armadillo repeat containing (*THADA*), which stands for thyroid adenoma-associated gene (**Table 1**) (74). This gene participates in metabolism and energy storage through the calcium pathway. In cancer, not only fusions but also polymorphisms are associated with the disease development (88). Particularly, it has been described that *THADA* is necessary to retain *CD27*4 in the Golgi for maturation. On the contrary, if suppressed, the immune response is triggered through the infiltration of *CD8* + T cells and increased toxicity (89). In addition to this finding, the *IGF2BP3-THADA* fusion has been demonstrated to cause overexpression of the partner gene *IGF2BP3*, leading to sustained growth and invasion through the activation of PI3KCA and MAPK pathways (34, 90). For its part, insulin-like growth factor 2 mRNA-binding protein 3 (*IGF2BP3*) is associated with a poor prognosis implicated in several mechanisms leading to aberrant metabolism in cancer (91). Currently, there are no inhibitors for *THADA*; however, the data strongly point out *THADA* as a potential therapeutic target in TC.

Another gene found in 30%–35% of FTC is PAX8-*PPARG*, characterized as an oncogene due to its binding to several genomic regions that code for genes related to cell proliferation, apoptosis evasion, and motility (92). Contrary to the case of repurposing losartan to *PAX8* alterations, this fusion promotes tumor progression due to the likely loss of functions of peroxisome proliferator-activated receptor gamma (*PPARG*). When inhibited with pioglitazone, anti-inflammatory effects and growth modulation are observed; however, the function of the gene fusion is not yet fully understood (35). Opposed to this idea, the antitumoral effect of *PPARG* inhibitor GW9662 has also been described, indicating the existence of independent pathways of *PPARG* (93). Remarkably, *PAX8-PPARG* is not the only fusion in TC involving *PPARG*; there is also *CREB3L2-PPARG* (94).

*NUT-BDR4* is an oncogenic driver fusion that causes a rare type of cancer named NUT midline carcinoma. Bromodomain-containing protein 4 (*BDR4*) binds to the chromatin, while *NUT* midline carcinoma family member 1 (*NUT*) recruits histone acetyltransferase (*HAT*), promoting the expression of several associated oncogenes (95). This rare fusion has also been described in some TC cases, and it is associated with high expression of *CD274* (96). The prognosis is low, with an estimated overall survival of 10 months, while therapy consists of radiotherapy and standard chemotherapy for large tumors. With no targeted therapy available, it is an urgent matter to start studying potential inhibitors for the treatment of these patients (97). Currently, only one inhibitor has been proposed to target the NUT-*BDR4* fusion. It consists of a dual inhibitor of bromodomain and extra-terminal motif (*BET*) proteins and the p300 bromodomain, named NEO2734, with proven inhibition of tumor growth and improvement of overall survival (36).

Lastly, *MET* not only has point mutations in TC but also a gene fusion. It has been identified that *TGF-MET* fusion is present in sarcoma, glioma, and TC (37, 98). Interestingly, in sarcoma, tumors that have this variant do not fit into a specific subtype, which is a remarkable finding due to the existence of effective *MET* inhibitors (50, 98).

## Conclusions and perspectives

It is relevant to recognize that in the era of personalized medicine, drug repositioning has a major impact on oncology. This is possible due to the identification of new therapeutic targets, which can be shared in different diseases and even between cancers. This opens a whole window of opportunity for the use of a plethora of drugs, reducing the time and costs involved in the production of new drugs, which has a positive impact on patients. In this review, we found that several drugs used in different types of cancer can be repositioned to TC, either by the presence of point mutations or by gene fusions. We found an area of opportunity for 13 genes with missense mutations and 10 for gene fusions. Among all these drugs, 22 are FDA-approved drugs, while the remaining five are inhibitors with proven efficacy in *in vitro* studies, both of which represent a promising area of therapy opportunity. It is the aim of this work to highlight the relevance of the identification of new potential inhibitors for genes that are part of recurrent fusion formation in TC as well as other types of cancer due to the likelihood of their contribution to disease development. Hence, it is of interest to the clinic to elucidate these variants’ potential as biomarkers or prognostic or therapeutic targets.

## Author contributions

DS-M: Writing – review & editing, Writing – original draft, Conceptualization. MS-C: Investigation, Formal analysis, Writing – original draft. MG-DC: Writing – original draft, Investigation, Formal analysis. AC-P: Writing – review & editing, Supervision, Resources, Project administration, Methodology, Funding acquisition, Conceptualization, Writing – original draft, Investigation.
